# Supplementary material for: A UMLS-based spell checker for natural language processing in vaccine safety
Source: BMC Med Inform Decis Mak. 2007 Feb 12;7:3. doi: 10.1186/1472-6947-7-3 (PMC1805499; doi:10.1186/1472-6947-7-3)
Supplement: Additional file 13 — RAP application directory. Sets up directory for RAP (RDF application for PHP) source code [file 1472-6947-7-3-S13.gz › rap/doc/phpdoc/__filesource/fsource_resModel__resModelResModel.php.html]

xml version="1.0" encoding="iso-8859-1"?


File Source for ResModel.php


# Source for file ResModel.php

Documentation is available at ResModel.php

```
1. <?php
2. // ----------------------------------------------------------------------------------
3. // Class: ResModel
4. // ----------------------------------------------------------------------------------


8. /**
9. * A ResModel provides an resource centric view on an underlying RDF model.
10. * ResModels show information not as statements but as resources with
11. * properties, similar to Jena models. ResModels may create Resources [URI
12. * nodes and bnodes]. Creating a Resource does not make the Resource visible to
13. * the model; Resources are only "in" Models if Statements about them are added
14. * to the Model. Similarly the only way to "remove" a Resource from a Model is
15. * to remove all the Statements that mention it.
16. *
17. * When a Resource or Literal is created by a Model, the Model is free to re-use an existing
18. * Resource or Literal object with the correct values, or it may create a fresh one.
19. *
20. * <BR><BR>History:
21. * <LI>09-10-2004                : First version of this class.</LI>
22. *
23. * @version  V0.9.1
24. * @author Daniel Westphal <mail at d-westphal dot de>
25. *
26. *
27. * @package     resModel
28. * @access    public
29. ***/

31. class ResModel
32. {

34. /**
35. * Holds a reference to the assoiated memmodel/dbmodel/infmodel
36. * @var        ResResource
37. * @access    private
38. */
39. var $model;


42. /**
43. * Constructor
44. * You have to supply a memmodel/dbmodel/infmodel to save the statements.
45. *
46. * @param object model $model
47. * @access    public
48. */
49. function ResModel(& $model)
50. {
51. if (!is_a($model,'Model'))
52. trigger_error(RDFAPI_ERROR . '(class: ResourceLayer; method: ResourceLayer):
53. $model has to be object of class Model', E_USER_ERROR);

55. $this->model =& $model;
56. }

58. /**
59. * Create a new resource associated with this model.
60. * If the uri string isn't set, this creates a bnode.
61. * Otherwise it creates a URI node.
62. * A URI resource is .equals() to any other URI Resource with the same URI
63. * (even in a different model - be warned).
64. *
65. * This method may return an existing Resource with the correct URI and model,
66. * or it may construct a fresh one, as it sees fit.
67. *
68. * Operations on the result Resource may change this model.
69. *
70. * @param    string    $uri
71. * @return    object ResResource
72. * @access    public
73. */
74. function createResource($uri = null)
75. {
76. $resResource = new ResResource($uri);
77. //associate the resource with this model, and get a unique identifier
78. //if it is bnode.
79. $resResource->setAssociatedModel($this);

81. return $resResource;
82. }

84. /**
85. * Create a new Property associated with this model.
86. * This method may return an existing property with the correct URI and model,
87. * or it may construct a fresh one, as it sees fit.
88. *
89. * Subsequent operations on the returned property may modify this model.
90. *
91. *
92. * @param    string    $uri
93. * @return    object ResProperty
94. * @access    public
95. */
96. function createProperty($uri = null)
97. {
98. $resProperty = new ResProperty($uri);
99. $resProperty->setAssociatedModel($this);

101. return $resProperty;
102. }

104. /**
105. * Create an untyped literal from a String value with a specified language.
106. *
107. * If you want to type this literal, you have to set a datatype before
108. * adding it to the model.
109. *
110. *
111. * @param    string    $label
112. * @param    string    $languageTag
113. * @return    object ResLiteral
114. * @access    public
115. */
116. function createLiteral($label,$languageTag = null)
117. {
118. $resLiteral = new ResLiteral($label,$languageTag);
119. $resLiteral->setAssociatedModel($this);

121. return $resLiteral;
122. }

124. /**
125. * General method to search for triples.
126. * NULL input for any parameter will match anything.
127. * Example:  $result = $m->find( NULL, NULL, $node );
128. * Finds all Statements with $node as object.
129. * Returns an array of statements with ResResources.
130. *
131. * @param    object ResResource    $subject
132. * @param    object ResResource    $predicate
133. * @param    object ResResource    $object
134. * @return    array
135. * @access    public
136. * @throws    PhpError
137. */
138. function find($subject,$predicate, $object)
139. {
140. $result=array();
141. //convert ResResources to Resources and Blanknodes
142. $resmodel=$this->model->find(    $this->_resNode2Node($subject),
143. $this->_resNode2Node($predicate),
144. $this->_resNode2Node($object)
145. );
146. //convert Resources, Blanknodes to ResResources
147. foreach ($resmodel->triples as $statement)
148. {
149. $result[]=new Statement($this->_node2ResNode($statement->getSubject()),
150. $this->_node2ResNode($statement->getPredicate(),true),
151. $this->_node2ResNode($statement->getObject())
152. );
153. };
154. return $result;

156. }

158. /**
159. * Searches for triples and returns the first matching statement.
160. * NULL input for any parameter will match anything.
161. * Example:  $result = $m->findFirstMatchingStatement( NULL, NULL, $node );
162. * Returns the first statement with ResResources of the Model where the object equals $node.
163. * Returns an NULL if nothing is found.
164. * You can define an offset to search.
165. *
166. * @param    object Node    $subject
167. * @param    object Node    $predicate
168. * @param    object Node    $object
169. * @param    integer $offset
170. * @return    object Statement
171. * @access    public
172. */
173. function findFirstMatchingStatement($subject,$predicate,$object,$offset = 0)
174. {

176. $statement = $this->model->findFirstMatchingStatement(    $this->_resNode2Node($subject),
177. $this->_resNode2Node($predicate),
178. $this->_resNode2Node($object),
179. $offset
180. );
181. if ($statement!==null)
182. {
183. return new Statement(    $this->_node2ResNode($statement->getSubject()),
184. $this->_node2ResNode($statement->getPredicate(),true),
185. $this->_node2ResNode($statement->getObject())
186. );
187. } else
188. {
189. return null;
190. }
191. }

193. /**
194. * Adds a new triple to the Model without checking if the statement is already in the Model.
195. * So if you want a duplicate free Model use the addWithoutDuplicates() function (which is slower then add())
196. * Expects a statements with ResResources(ResLiterals)
197. *
198. * @param    object Statement    $statement
199. * @access    public
200. * @throws    PhpError
201. */
202. function add($statement)
203. {
204. return $this->model->add(new Statement(    $this->_resNode2Node($statement->getSubject()),
205. $this->_resNode2Node($statement->getPredicate()),
206. $this->_resNode2Node($statement->getObject()))
207. );
208. }
209. /**
210. * Checks if a new statement is already in the Model and adds the statement, if it is not in the Model.
211. * addWithoutDuplicates() is significantly slower then add().
212. * Retruns TRUE if the statement is added.
213. * FALSE otherwise.
214. * Expects a statements with ResResources(ResLiterals)
215. *
216. * @param    object Statement    $statement
217. * @return   boolean
218. * @access    public
219. * @throws    PhpError
220. */
221. function addWithoutDuplicates($statement)
222. {
223. return $this->model->addWithoutDuplicates(new Statement($this->_resNode2Node($statement->getSubject()),
224. $this->_resNode2Node($statement->getPredicate()),
225. $this->_resNode2Node($statement->getObject()))
226. );
227. }

229. /**
230. * Tests if the Model contains the given statement.
231. * TRUE if the statement belongs to the model;
232. * FALSE otherwise.
233. * Expects a statement of ResResources(ResLiterals)
234. *
235. * @param    object Statement    $statement
236. * @return    boolean
237. * @access    public
238. */
239. function contains(& $statement)
240. {

242. return $this->model->contains(new Statement($this->_resNode2Node($statement->getSubject()),
243. $this->_resNode2Node($statement->getPredicate()),
244. $this->_resNode2Node($statement->getObject()))
245. );
246. }

248. /**
249. * Determine if all of the statements in a model are also contained in this model.
250. * True if all of the statements in $model are also contained in this model and false otherwise.
251. *
252. * @param    object Model    &$model
253. * @return    boolean
254. * @access    public
255. */
256. function containsAll(& $model)
257. {
258. if (is_a($model,'ResModel'))
259. return $this->model->containsAll($model->getModel());

261. return $this->model->containsAll($model);
262. }

264. /**
265. * Determine if any of the statements in a model are also contained in this model.
266. * True if any of the statements in $model are also contained in this model and false otherwise.
267. *
268. * @param    object Model    &$model
269. * @return    boolean
270. * @access    public
271. */
272. function containsAny(& $model)
273. {
274. if (is_a($model,'ResModel'))
275. return $this->model->containsAny($model->getModel());
276. return $this->model->containsAny($model);
277. }

279. /**
280. * Determine if the node (ResResource / ResLiteral) $node appears in any statement of this model.
281. *
282. * @param    object Node    &$node
283. * @return    boolean
284. * @access    public
285. */
286. function containsResource(& $node)
287. {
288. if ($this->findFirstMatchingStatement($node,null,null) === null)
289. if ($this->findFirstMatchingStatement(null,$node,null) === null)
290. if ($this->findFirstMatchingStatement(null,null,$node) === null)
291. return false;

293. return true;
294. }

296. /**
297. * Create a literal from a String value with the $dtype Datatype
298. * An existing literal of the right value may be returned, or a fresh one created.
299. *
300. * @param    string    $value
301. * @param    string     $dtype
302. * @return     object ResLiteral
303. * @access    public
304. */
305. function createTypedLiteral($value,$dtype)
306. {
307. $resLiteral = new ResLiteral($label);
308. $resLiteral->setDatatype($dtype);
309. $resLiteral->setAssociatedModel($this);

311. return $resLiteral;
312. }

314. /**
315. * Checks if two models are equal.
316. * Two models are equal if and only if the two RDF graphs they represent are isomorphic.
317. *
318. * Warning: This method doesn't work correct with models where the same blank node has different
319. * identifiers in the two models. We will correct this in a future version.
320. *
321. * @access    public
322. * @param    object    model &$that
323. * @throws    phpErrpr
324. * @return    boolean
325. */
326. function equals(& $that)
327. {
328. if (is_a($that,'ResModel'))
329. return $this->model->equals($that->getModel());
330. return $this->model->equals($that);
331. }

333. /**
334. * Returns a new model that is the subtraction of another model from this model.
335. *
336. * @param    object Model $model
337. * @return    object MemModel
338. * @access    public
339. * @throws phpErrpr
340. */
341. function subtract($model)
342. {
343. if (is_a($model,'ResModel'))
344. return $this->model->subtract($model->getModel());
345. return $this->model->subtract($model);
346. }

348. /**
349. * Answer a statement find(s, p, null) with ResResources(ResLiterals) from this model.
350. * If none exist, return null; if several exist, pick one arbitrarily.
351. *
352. * @param    object ResResource $subject
353. * @param    object ResResource $property
354. * @return    object Statement
355. * @access    public
356. * @throws phpErrpr
357. */
358. function getProperty($subject,$property)
359. {

361. $statement= $this->model->findFirstMatchingStatement(    $this->_resNode2Node($subject),
362. $this->_resNode2Node($property),
363. null
364. );
365. if ($statement === null)
366. return null;

368. return new Statement($this->_node2ResNode($statement->getSubject()),
369. $this->_node2ResNode($statement->getPredicate(),true),
370. $this->_node2ResNode($statement->getObject())
371. );

373. }

375. /**
376. * Checks if MemModel is empty
377. *
378. * @return    boolean
379. * @access    public
380. */
381. function isEmpty()
382. {
383. return $this->model->isEmpty();
384. }

386. /**
387. * Returns a ResIterator with all objects in a model.
388. *
389. * @return    object ResIterator
390. * @access    public
391. * @throws phpErrpr
392. */
393. function listObjects()
394. {
395. return $this->listObjectsOfProperty(null);
396. }

398. /**
399. * Returns a ResIterator with all objects with a given property and property value.
400. *
401. * @param    object ResResource    $property
402. * @param    object ResResource    $value
403. * @return    object ResIterator
404. * @access    public
405. */
406. function listObjectsOfProperty($property, $value = null)
407. {
408. return new ResIterator(null,$property,$value,'o',$this);
409. }


412. /**
413. * Returns a ResIterator with all subjects in a model.
414. *
415. * @return    object ResIterator
416. * @access    public
417. * @throws phpErrpr
418. */
419. function listSubjects()
420. {
421. return $this->listSubjectsWithProperty(null);
422. }

424. /**
425. * Returns a ResIterator with all subjects with a given property and property value.
426. *
427. * @param    object ResResource    $property
428. * @param    object ResResource    $value
429. * @return    object ResIterator
430. * @access    public
431. * @throws phpErrpr
432. */
433. function listSubjectsWithProperty($property,$value = null)
434. {
435. return new ResIterator(null,$property,$value,'s',$this);
436. }

438. /**
439. * Removes the statement of ResResources(ResTriples) from the MemModel.
440. * TRUE if the statement is removed.
441. * FALSE otherwise.
442. *
443. * @param    object Statement    $statement
444. * @return   boolean
445. * @access    public
446. * @throws    PhpError
447. */
448. function remove($statement)
449. {
450. return $this->model->remove(new Statement(    $this->_resNode2Node($statement->getSubject()),
451. $this->_resNode2Node($statement->getPredicate()),
452. $this->_resNode2Node($statement->getObject())
453. ));
454. }

456. /**
457. * Number of statements in the MemModel
458. *
459. * @return    integer
460. * @access    public
461. */
462. function size()
463. {
464. return $this->model->size();
465. }

467. /**
468. * Returns a new Model that is the set-union of the model with another model.
469. * Duplicate statements are removed. If you want to allow duplicates, use addModel() which is much faster.
470. *
471. * The result of taking the set-union of two or more RDF graphs (i.e. sets of triples)
472. * is another graph, which we will call the merge of the graphs.
473. * Each of the original graphs is a subgraph of the merged graph. Notice that when forming
474. * a merged graph, two occurrences of a given uriref or literal as nodes in two different
475. * graphs become a single node in the union graph (since by definition they are the same
476. * uriref or literal) but blank nodes are not 'merged' in this way; and arcs are of course
477. * never merged. In particular, this means that every blank node in a merged graph can be
478. * identified as coming from one particular graph in the original set of graphs.
479. *
480. * Notice that one does not, in general, obtain the merge of a set of graphs by concatenating
481. * their corresponding N-triples documents and constructing the graph described by the merged
482. * document, since if some of the documents use the same node identifiers, the merged document
483. * will describe a graph in which some of the blank nodes have been 'accidentally' merged.
484. * To merge Ntriples documents it is necessary to check if the same nodeID is used in two or
485. * more documents, and to replace it with a distinct nodeID in each of them, before merging the
486. * documents. (Not implemented yet !!!!!!!!!!!)
487. *
488. * @param    object Model    $model
489. * @return    object MemModel
490. * @access    public
491. * @throws phpErrpr
492. *
493. */
494. function & unite(& $model)
495. {
496. if (is_a($model,'ResModel'))
497. return $this->model->unite($model->getModel());
498. return $this->model->unite($model);
499. }

501. /**
502. * Adds another model to this MemModel.
503. * Duplicate statements are not removed.
504. * If you don't want duplicates, use unite().
505. * If any statement of the model to be added to this model contains a blankNode
506. * with an identifier already existing in this model, a new blankNode is generated.
507. *
508. * @param    object Model    $model
509. * @access    public
510. * @throws phpErrpr
511. *
512. */
513. function addModel(&$model)
514. {
515. if (is_a($model,'ResModel'))
516. return $this->model->addModel($model->getModel());
517. return $this->model->addModel($model);
518. }

520. /**
521. * Create a new RDF Container from type rdf:Alt
522. * This method may return an existing container with the correct URI and model,
523. * or it may construct a fresh one, as it sees fit.
524. *
525. * Subsequent operations on the returned Container may modify this model.
526. *
527. *
528. * @param    string    $uri
529. * @return    object ResProperty
530. * @access    public
531. */
532. function createAlt($uri = null)
533. {
534. $resAlt = new ResAlt($uri);
535. $resAlt->setAssociatedModel($this);

537. return $resAlt;
538. }

540. /**
541. * Create a new RDF Container from type rdf:Bag
542. * This method may return an existing container with the correct URI and model,
543. * or it may construct a fresh one, as it sees fit.
544. *
545. * Subsequent operations on the returned Container may modify this model.
546. *
547. *
548. * @param    string    $uri
549. * @return    object ResProperty
550. * @access    public
551. */
552. function createBag($uri = null)
553. {
554. $resBag = new ResBag($uri);
555. $resBag->setAssociatedModel($this);

557. return $resBag;
558. }

560. /**
561. * Create a new RDF Container from type rdf:Seq
562. * This method may return an existing container with the correct URI and model,
563. * or it may construct a fresh one, as it sees fit.
564. *
565. * Subsequent operations on the returned Container may modify this model.
566. *
567. *
568. * @param    string    $uri
569. * @return    object ResProperty
570. * @access    public
571. */
572. function createSeq($uri = null)
573. {
574. $resSeq = new ResSeq($uri);
575. $resSeq->setAssociatedModel($this);

577. return $resSeq;
578. }

580. /**
581. * Create a new RDF Collection from type rdf:List
582. * This method may return an existing container with the correct URI and model,
583. * or it may construct a fresh one, as it sees fit.
584. *
585. * Subsequent operations on the returned Container may modify this model.
586. *
587. *
588. * @param    string    $uri
589. * @return    object ResProperty
590. * @access    public
591. */
592. function createList($uri = null)
593. {
594. $resList = new ResList($uri);
595. $resList->setAssociatedModel($this);

597. return $resList;
598. }

600. /**
601. * Returns a reference to the underlying model (Mem/DB/InfModel) that contains the statements
602. *
603. *
604. * @return    object Model
605. * @access    public
606. */
607. function & getModel()
608. {
609. return  $this->model;
610. }


613. /**
614. * Internal method, that returns a resource URI that is unique for the Model.
615. * URIs are generated using the base_uri of the Model, the prefix and a unique number.
616. * If no prefix is defined, the bNode prefix, defined in constants.php, is used.
617. *
618. * @param    string    $prefix
619. * @return    string
620. * @access    private
621. */
622. function getUniqueResourceURI($bnodePrefix)
623. {
624. return $this->model->getUniqueResourceURI($bnodePrefix);
625. }

627. /**
628. * Load a model from a file containing RDF, N3 or N-Triples.
629. * This function recognizes the suffix of the filename (.n3 or .rdf) and
630. * calls a suitable parser, if no $type is given as string ("rdf" "n3" "nt");
631. * If the model is not empty, the contents of the file is added to this DbModel.
632. *
633. * @param     string     $filename
634. * @param     string     $type
635. * @param   boolean $stream
636. * @access    public
637. */
638. function load($filename, $type = NULL, $stream=false)
639. {
640. $this->model->load($filename, $type = NULL, $stream=false);
641. }

643. /**
644. * Return current baseURI.
645. *
646. * @return  string
647. * @access    public
648. */
649. function getBaseURI()
650. {
651. return $this->model->getBaseURI();
652. }

654. /**
655. * Saves the RDF,N3 or N-Triple serialization of the MemModel to a file.
656. * You can decide to which format the model should be serialized by using a
657. * corresponding suffix-string as $type parameter. If no $type parameter
658. * is placed this method will serialize the model to XML/RDF format.
659. * Returns FALSE if the MemModel couldn't be saved to the file.
660. *
661. * @access    public
662. * @param     string     $filename
663. * @param     string     $type
664. * @throw   PhpError
665. * @return    boolean
666. */
667. function saveAs($filename, $type ='rdf')
668. {
669. return $this->model->saveAs($filename, $type ='rdf');
670. }

672. /**
673. * Writes the RDF serialization of the MemModel as HTML table.
674. *
675. * @access    public
676. */
677. function writeAsHTMLTable()
678. {
679. $this->model->writeAsHtmlTable();
680. }

682. /**
683. * Returns a new model containing all the statements which are in both this model and another.
684. *
685. * @param    object Model    $model
686. * @return    object MemModel
687. * @access    public
688. * @throws phpErrpr
689. */
690. function & intersect(& $model)
691. {
692. if (is_a($model,'ResModel'))
693. return $this->model->intersect($model->getModel());
694. return $this->model->intersect($model);
695. }

697. /**
698. * converts a Resource,Blanknode,Literal into a ResResource, ResProperty, or ResLiteral
699. *
700. * @param    object Node    $node
701. * @param    boolean        $isProperty
702. * @return    object ResResource / ResProperty / ResLiteral
703. * @access    private
704. * @throws phpErrpr
705. */
706. function _node2ResNode($node, $isProperty = false)
707. {
708. if (is_a($node,'Literal'))
709. {
710. $return= new ResLiteral($node->getLabel(),$node->getLanguage());
711. $return->setDatatype($node->getDatatype());
712. $return->setAssociatedModel($this);

714. return $return;
715. }
716. if (is_a($node,'Resource'))
717. {
718. if ($isProperty)
719. {
720. $res= new ResProperty($node->getLabel());
721. } else
722. {
723. $res= new ResResource($node->getLabel());
724. }
725. $res->setAssociatedModel($this);
726. if (is_a($node,'Blanknode'))
727. $res->setIsAnon(true);

729. return $res;
730. }
731. }

733. /**
734. * converts a ResResource, ResProperty, or ResLiteral into a Resource, Blanknode, or Literal
735. *
736. * @param    object ResNode    $resNode
737. * @return    object Node
738. * @access    private
739. * @throws phpErrpr
740. */
741. function _resNode2Node($resNode)
742. {
743. if (is_a($resNode,'ResResource'))
744. {
745. if ($resNode->getIsAnon())
746. {
747. $return=new BlankNode($resNode->getURI());
748. } else
749. {
750. $return=new Resource($resNode->getURI());
751. }
752. return $return;
753. }

755. if (is_a($resNode,'ResLiteral'))
756. {
757. $literal=new Literal($resNode->getLabel(),$resNode->getLanguage());
758. if ($resNode->getDatatype() != null)
759. $literal->setDatatype($resNode->getDatatype());
760. return $literal;
761. }
762. }

764. /**
765. * Set a base URI for the MemModel.
766. * Affects creating of new resources and serialization syntax.
767. * If the URI doesn't end with # : or /, then a # is added to the URI.
768. * @param    string    $uri
769. * @access    public
770. */
771. function setBaseURI($uri)
772. {
773. $this->model->setBaseURI($uri);
774. }

776. /**
777. * Writes the RDF serialization of the MemModel as HTML table.
778. *
779. * @access    public
780. * @return    string
781. */
782. function writeRdfToString()
783. {
784. $this->model->writeRdfToString();
785. }

787. /**
788. * Perform an RDQL query on this MemModel.
789. * This method returns an associative array of variable bindings.
790. * The values of the query variables can either be RAP's objects (instances of Node)
791. * if $returnNodes set to TRUE, or their string serialization.
792. *
793. * @access    public
794. * @param string $queryString
795. * @param boolean $returnNodes
796. * @return  array   [][?VARNAME] = object Node  (if $returnNodes = TRUE)
797. *       OR  array   [][?VARNAME] = string
798. *
799. */
800. function & rdqlQuery($queryString, $returnNodes = TRUE)
801. {
802. return $this->model->rdqlQuery($queryString, $returnNodes);
803. }

805. /**
806. * Perform an RDQL query on this MemModel.
807. * This method returns an RdqlResultIterator of variable bindings.
808. * The values of the query variables can either be RAP's objects (instances of Node)
809. * if $returnNodes set to TRUE, or their string serialization.
810. *
811. * @access    public
812. * @param string $queryString
813. * @param boolean $returnNodes
814. * @return  object RdqlResultIterator = with values as object Node  (if $returnNodes = TRUE)
815. *       OR  object RdqlResultIterator = with values as strings if (if $returnNodes = FALSE)
816. *
817. */
818. function rdqlQueryAsIterator($queryString, $returnNodes = TRUE)
819. {
820. return $this->model->rdqlQueryAsIterator($queryString, $returnNodes);
821. }


824. /**
825. * Returns the models namespaces.
826. *
827. * @author   Tobias Gau� <tobias.gauss@web.de>
828. * @access   public
829. * @return   Array
830. */
831. function getParsedNamespaces(){
832. return $this->model->getParsedNamespaces();
833. }


837. /**
838. * Adds the namespaces to the model. This method is called by
839. * the parser. !!!! addParsedNamespaces() not overwrites manual
840. * added namespaces in the model !!!!
841. *
842. * @author   Tobias Gau� <tobias.gauss@web.de>
843. * @access   public
844. * @param    Array $newNs
845. */
846. function addParsedNamespaces($newNs){
847. $this->model->addParsedNamespaces($newNs);
848. }


851. /**
852. * Adds a namespace and prefix to the model.
853. *
854. * @author   Tobias Gau� <tobias.gauss@web.de>
855. * @access   public
856. * @param    String $prefix, String $nmsp
857. */
858. function addNamespace($prefix, $namespace){
859. $this->model->addNamespace($prefix, $namespace);
860. }

862. /**
863. * removes a single namespace from the model
864. *
865. * @author   Tobias Gau� <tobias.gauss@web.de>
866. * @access   public
867. * @param    String $nmsp
868. */
869. function removeNamespace($nmsp){
870. return $this->model->removeNamespace($nmsp);
871. }


878. }
```

Documentation generated on Thu, 7 Jul 2005 13:43:43 +0200 by phpDocumentor 1.3.0RC3
